# Open Reduction and Internal Fixation and Cement-In-Cement Revision for Selected Vancouver B Proximal Femur Periprosthetic Fractures

**DOI:** 10.1016/j.artd.2022.101071

**Published:** 2022-12-12

**Authors:** Cathal J. McCarthy, Joss Moore, Lauren Tiedt, Finbarr Condon

**Affiliations:** a Department of Trauma and Orthopaedics, University Hospital Limerick, Limerick, Ireland; b University of Limerick, County Limerick, Ireland

**Keywords:** Arthroplasty, Hip, Periprosthetic, Cement, Osteoporosis

## Abstract

The incidence of periprosthetic proximal femoral fractures is increasing with the increase in arthroplasty being performed as well as aging populations. We describe an open reduction and internal fixation and cement-in-cement technique utilizing a well-fixed cement mantle. The advantages of this allow for a shorter operative time, reduction in risk of iatrogenic femoral fractures, and reduction in blood loss. This was a retrospective study reviewing 20 patients that underwent this technique for periprosthetic fractures. Thirty percent (n = 6) of patients underwent subsequent surgery. We had a 95% (n = 19) union rate with 1 case refracturing through the old fracture. This technique can allow for shorter operative times and a lower physiological insult in reducible periprosthetic proximal femur fractures with a stable cement mantle.

## Introduction

As the incidence of total hip arthroplasty (THA) increases as well as the increase in age of the population, the rate of periprosthetic femoral fractures has been increasing [[1], [2], [3]]. These are challenging injuries to manage that can result in significant morbidity for patients and deterioration in patients’ functional outcomes. It was found in the United Kingdom that there was a 13% annual rise in periprosthetic femoral fractures between April 2015 and December 2018 with a 3.3% 1-month mortality in these patients [4].

In our study, we describe a cement-in-cement (c-in-c) revision technique for periprosthetic fractures that can be utilized in B1 and B2 fractures according to the Vancouver Classification [5]. However, we would argue that B1-type fractures do not occur around polished tapered stems, as they are always loose at surgery and result in what has been described as an “axe-splitting” mechanism of fracture [6,7]. Revision of periprosthetic fractures is a complex operation where removing the existing cement mantle is usually a very difficult task that can prove to be time-consuming and can result in complications such as further fracture of the femur in already vulnerable patients.

We describe a c-in-c technique where a well-fixed cement mantle may be used, with the insertion of a smaller stem than the original, without needing to modify the original cement mantle in any way. The advantages of this allow for a shorter operative time, reduction of iatrogenic femoral fracture risk, and reduction in blood loss.

The c-in-c technique has been well described for its use in revision arthroplasty in other settings [[8], [9], [10], [11], [12]], but little has been described regarding its use in periprosthetic fractures. Woodbridge et al. [9] performed a follow-up study on their c-in-c experience with the Exeter short revision stem (Stryker, Mahwah, NJ), and of 166 cases that they performed, only 1 was for a periprosthetic fracture.

## Methods

This was a retrospective review of 20 patients that underwent c-in-c revision for Vancouver B periprosthetic proximal femur fractures between September 2012 and May 2021. The mean age of patients in this group was 77 years (range 60 – 87). There were 14 men and 6 women that underwent a c-in-c revision surgery for a periprosthetic fracture.

Theater logbooks over a 10-year period were searched for all periprosthetic fractures that were managed in our unit, operative notes were reviewed, and patients that underwent open reduction and internal fixation (ORIF) and c-in-c revision for their management were selected. Patients were selected for this method by the treating surgeon based on the appearance of the fracture radiographically and intraoperatively after inspection of the cement mantle after fracture reduction. The decision to use c-in-c revision was also based on patient factors such as age, comorbidities, and baseline function for the treatment method felt to provide the best outcome. Excluded were any patients that did not undergo c-in-c revision due to fractures around uncemented stems, where fractures had inadequate cement mantle or bone stock (Vancouver B3) to perform c-in-c technique and fractures below the stem (Vancouver C). Images were reviewed to assess the type of fracture that had occurred and to confirm the operative intervention. The National Integrated Medical Imaging System was used to assess for any further revisions that occurred in these patients, as well as for searching our own operative logbooks for revisions in our unit.

Patient charts were reviewed, and we obtained American Society of Anaesthesiologist (ASA) scores, Charlson Comorbidity Index [13] scores, and operative times. Patients were searched on the patient registration system of the hospital to assess mortality.

All patients had postoperative radiographs performed the day of the surgery, and sequential follow-up radiographs were also reviewed.

Institutional review board approval was not obtained as this was a retrospective audit of clinical records which were already obtained.

Surgeries were performed between two fellowship-trained arthroplasty surgeons in our unit.

### Surgical technique

**Figure 1:**
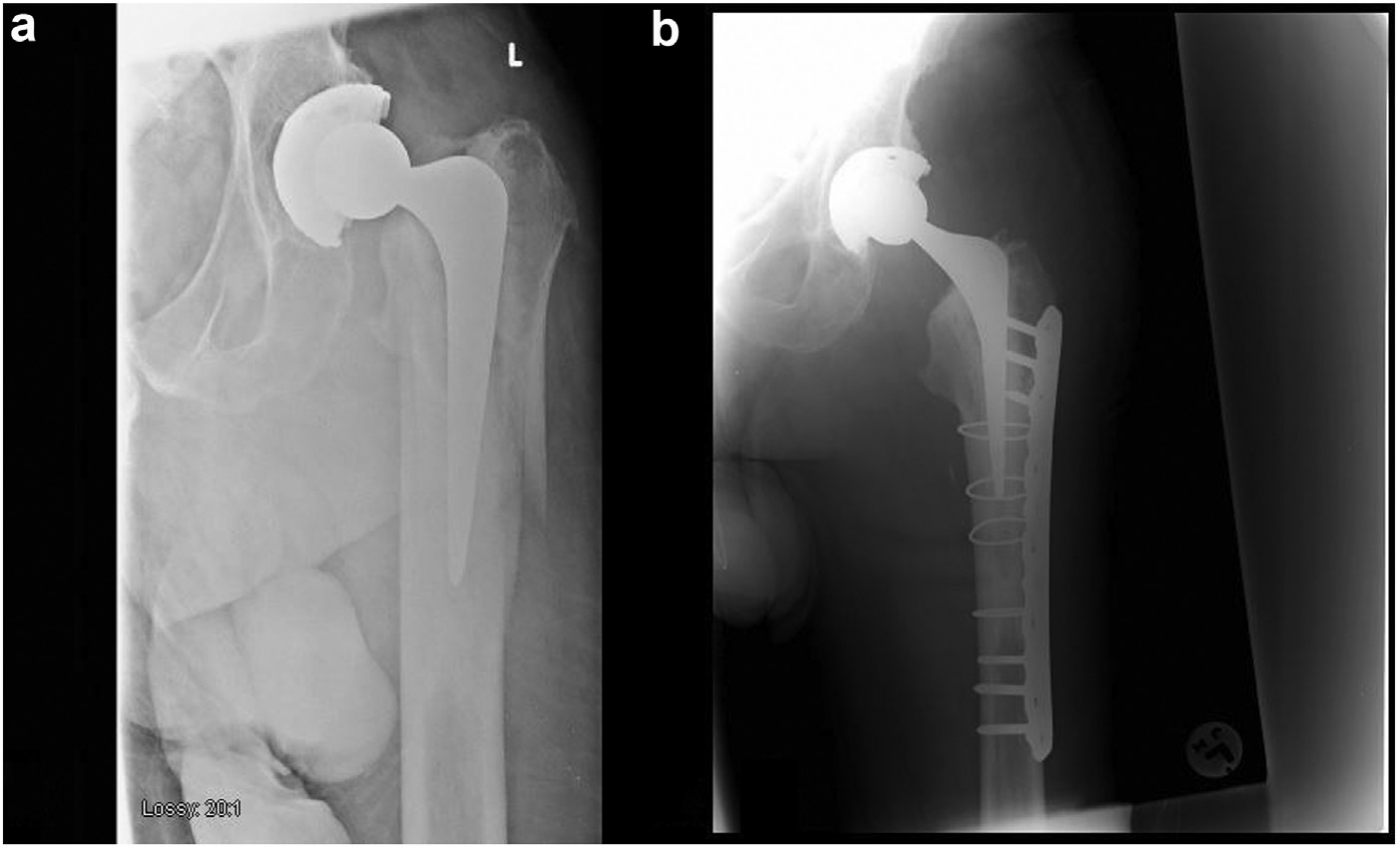
(a) Periprosthetic fracture in a total hip arthroplasty. (b) The same patient after undergoing cement-in-cement with ORIF revision to a short Exeter stem.

**Figure 2:**
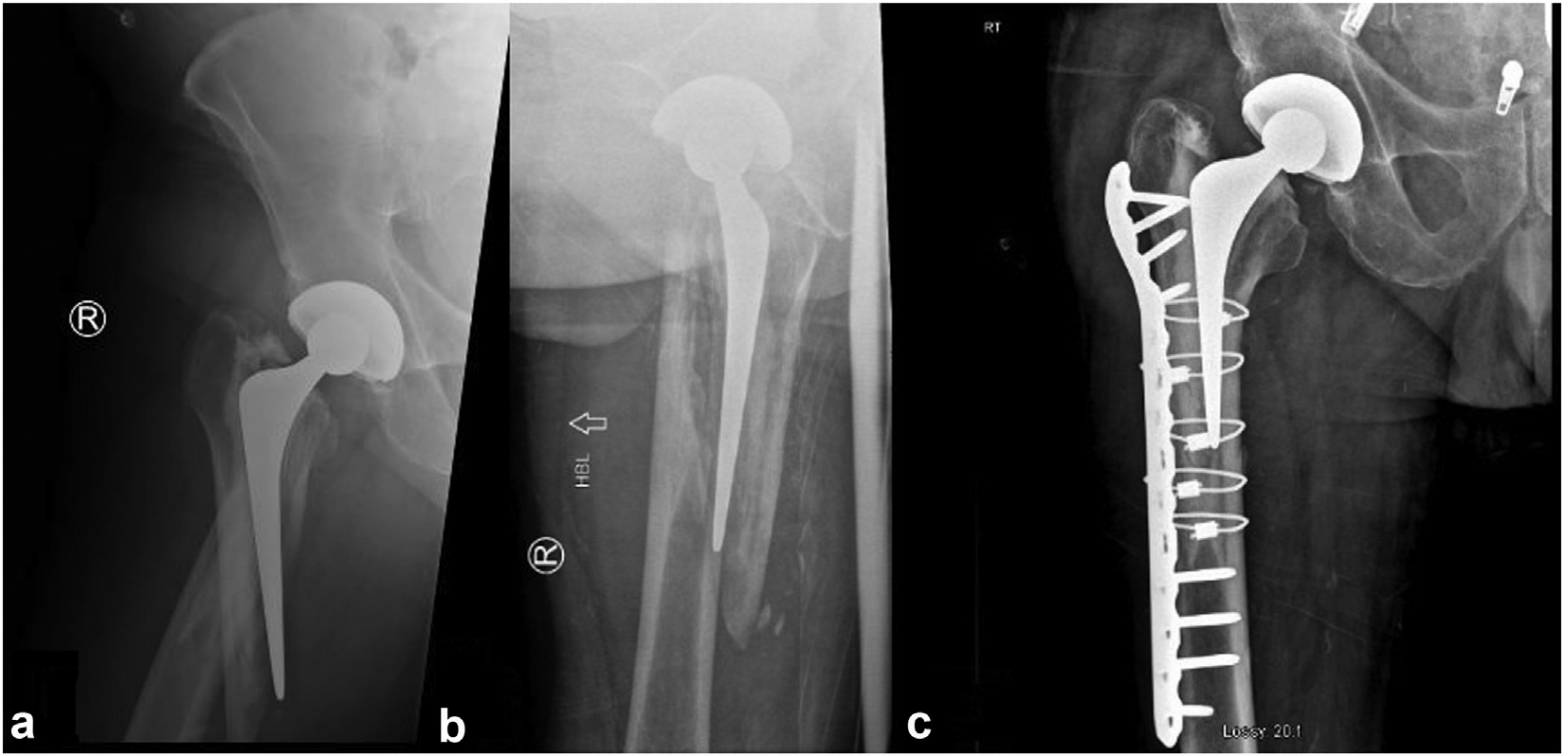
(a) Anteroposterior radiograph of the periprosthetic fracture in a THA. (b) Vertical orientation of the cross table lateral radiograph. (c) The same patient after cement-in-cement with ORIF revision to a short Exeter stem.

Patients were positioned in a lateral decubitus position, with spinal anesthesia usually being used. Antimicrobial prophylaxis was used with 1.5 g of cefuroxime. An extensile posterior surgical approach was used to the hip and femur to allow for dislocation of the hip, removal of the stem, and satisfactory reduction of the fracture. Adequate exposure is important to make assessment of the fracture pattern and cement suitability intraoperatively and to allow for anatomical fracture reduction, a key component for this technique. The cement mantle in Gruen zone 1 [14] was curetted out using a burr to allow for safe stem removal. Collarless, polished, straight-tapered stems are more easily removed from the cement mantle, without damaging the cement mantle. The fracture was then reduced initially using braided cables. Once the cement mantle was reduced along with the fracture, it was inspected closely with the aid of a narrow cable light source. If it was found to be intact below the lesser trochanter, it was deemed suitable for the c-in-c technique. Consideration was also given for revision of the cup and/or liner due to the risk of dislocation in this cohort. A stem smaller than the previous stem should be used to allow for c-in-c implantation. We often used the Exeter short revision stem, which has an offset of 44 mm and a length of 125 mm compared with the standard Exeter stem which has a length of 150 mm. The Exeter short revision stem is also slimmer front to back than the standard Exeter stems. If the version of the mantle was unsatisfactory, we used a burr to the cement mantle proximally to allow a change of version from the old stem. The cement mantle was then washed and dried. Polymethylmethacrylate bone cement (PMMA) was placed into the cement mantle in a retrograde fashion and pressurized to allow the PMMA to propagate into any cracks, fissures, and any exposed bone. Adjunct extramedullary fixation was then performed with combinations of locked plates, cortical onlay strut allograft with morselized femoral head allograft in its concavity, and further cabling which bypassed the fracture site. See Figure 1, Figure 2 for examples of patients that underwent this surgical procedure.

### Statistical analysis

Data were collected using Microsoft Excel 365 (Microsoft Corporation, Redmond, WA). Data analysis was performed using Statistical Package for the Social Studies (SPSS) version 23 (IBM Corp., Armonk, NY).

## Results

According to the Vancouver classification [5], radiographically, there were 4 Vancouver B1 fracture patterns and 16 Vancouver B2 fracture patterns that underwent a c-in-c revision surgery. For the fracture patterns, there were 13 spiral fractures and 7 oblique fractures. Thirteen of the fractures occurred around a THA, and seven occurred around a hemiarthroplasty. There were 6 patients with an ASA score of 2, 12 with ASA 3, and 2 with ASA 4. The median Charlson Comorbidity Index score was 4.5 (range 3 -7), with all patients having a score of 3 or more. There was a 50% mortality, with the mean time to death in this group being 2.2 years (range 3 months – 7.1 years). The mean operative time was 129 minutes (range 80 – 208 minutes).

One patient in the THA group had their acetabular cup revised, and 1 patient in the hemiarthroplasty group was convinced to undergo a THA having an acetabular cup inserted with a constrained liner. Two patients of the THA group already had constrained liners in place, and neither of these patients had revision of their cup performed. Nineteen fractures occurred around Exeter stems, and 1 fracture occurred around a Charnley stem (Depuy, Warsaw, IN). Regarding the stem revisions, eight were revised to a standard Exeter stem, and twelve were revised to a short Exeter stem.

Thirteen patients had their original surgery performed in our unit. The mean time from surgery to injury of these patients was 2.8 years (range 6 months – 6 years). The mean time from injury to revision surgery was 4.5 days (range 1 – 9 days). The mean time from c-in-c revision surgery to repeat revision was 15 months (range 20 days – 5 years).

Six (30%) underwent a subsequent revision surgery. Four of these were related to dislocations. Three had revision of the liner to a constrained liner. One patient sustained a stem pullout of a standard Exeter stem while attempting a reduction and underwent stem revision with revision of the liner to a constrained liner.

**Figure 3:**
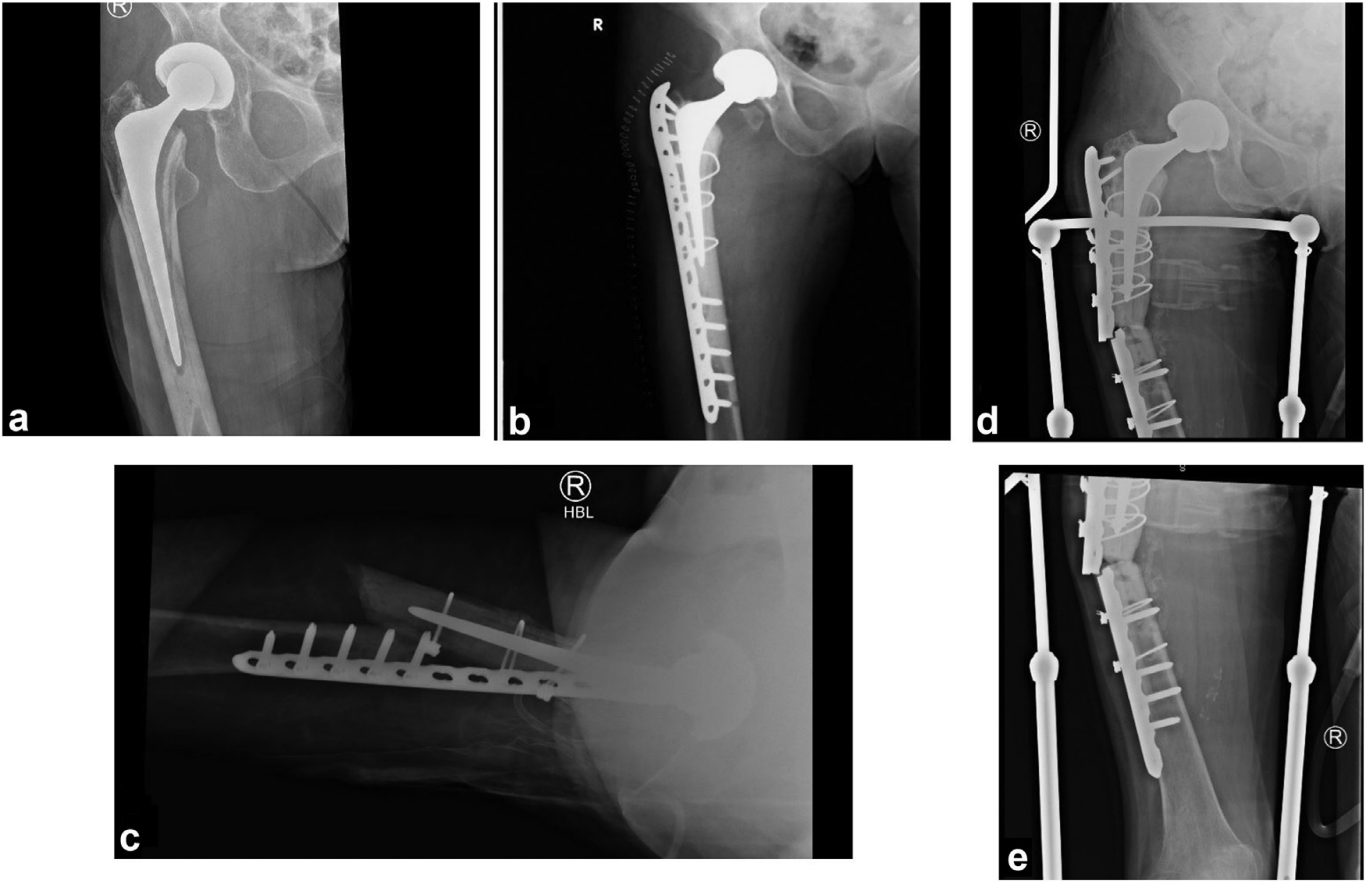
(a) Anteroposterior (AP) radiograph of the periprosthetic fracture in a THA. (b) The same patient after cement-in-cement revision to a short Exeter stem. (c) Cross-table lateral of refracture distal to the short Exeter stem. (d) AP hip of refracture after repeat ORIF and bone grafting. (e) AP distal femur of refracture after repeat ORIF and bone grafting.

**Figure 4:**
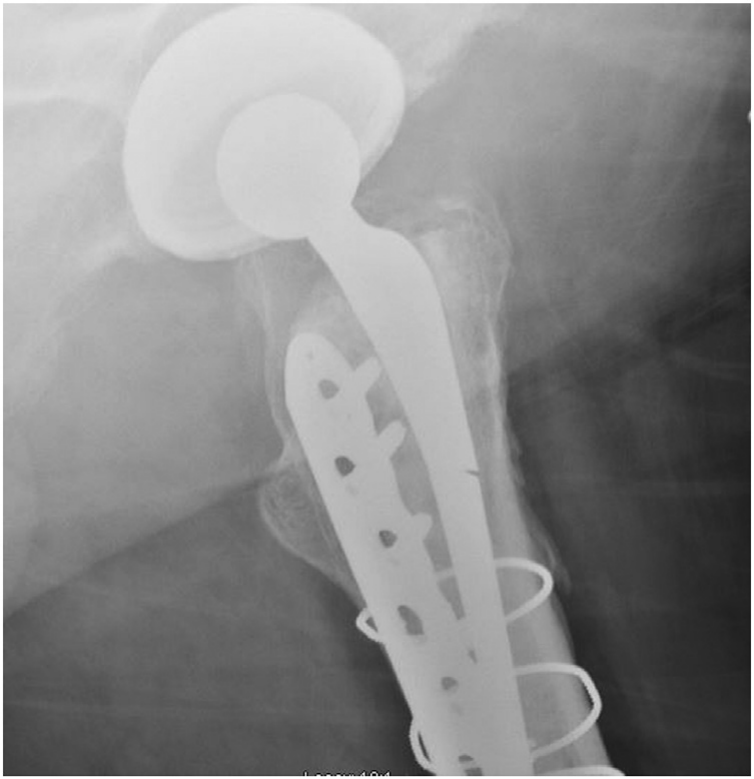
Lateral radiograph of the hip showing a stem fracture of a short Exeter stem.

There was 1 revision for a refracture through a nonunion. This refracture occurred around the tip of a short Exeter stem where the initial fracture occurred around the body of a standard Exeter stem. This patient underwent repeat ORIF with bone grafting. This patient then subsequently had another refracture and underwent proximal femur replacement with constrained liner placement. See Figure 3 for this patient’s operative course. There was 1 revision for a femoral stem fracture of the proximal body in a short Exeter stem which was subsequently converted to a diaphyseal bearing stem. See Figure 4 for the femoral stem fracture.

There were four (20%) dislocations in total which were all revised as previously discussed.

For the breakdown of methods of fixation performed, see Table 1.

**Table 1:** Methods of fixation.

Method of fixation	Number
Plate + cables + screws	13
Strut graft + plate + cables + screws	6
Strut graft + cables	1

## Discussion

The c-in-c revision technique offers a good option for revision of periprosthetic fractures in certain circumstances where it can allow for a faster surgery while minimizing the physiological insult in patients that have a lower demand where there is a reducible fracture with an intact cement mantle. We present a case series of a novel technique for the management of periprosthetic fractures, particularly around polished tapered stems. There is a paucity of published data in the literature of using this or similar techniques [[15], [16], [17]], which is a viable option in an elderly patient or patients with significant medical comorbidities where a prolonged complex surgery carries a high risk of morbidity and mortality.

Richards et al. [17] described their technique of c-in-c which is similar to ours. In their technique, they routinely used a rigid knee arthroscope to visualize the reduced cement mantle to verify that anatomical reduction of cement mantle has been achieved. While they described adjunct fixation options, they did not describe extramedullary fixation bypassing the fracture site which we perform to augment the fixation and add rotational stability. At the time of publishing their article, only 3 patients had undergone this technique, with all 3 patients uniting without issue and no revisions being performed.

Briant-Evans et al. [16] described a c-in-c technique for periprosthetic fractures that varies from ours. In their technique, they used a larger, longer stem than the original stem after debulking the distal cement mantle using a burr or power reamer. Their aim was to bypass the fracture site internally, which they achieved in 20 of 23 cases. In their technique, they used a strut graft or plate fixation in 11 of 23 cases. They used standard and long Exeter stems as part of their study, with the planning of which stem to use related to the stems' ability to bypass the fracture site. For the group of 20 that bypassed the fracture site, they did so at a mean of 2.5 cortical diameters. They had 1 nonunion case, which had a refracture with fracture through the plate.

We only had 1 patient (5%) that had a nonunion of the fracture. This patient underwent repeat ORIF and subsequently had a refracture again with fracture of the plate which was revised to a diaphyseal bearing stem. Including the other two studies on c-in-c revision for periprosthetic fractures, there were 2 nonunion cases in 46 patients (4.3%) [16,17].

In our study group, the short Exeter stem was significantly used (44/00/125), with twelve (60%) of our patients having this implant utilized as part of the c-in-c technique. The short Exeter stem provides a good option for a smaller stem which can be implanted into the old cement mantle. Richards et al. [17] described as part of their technique that those who do not have a short stem option available may consider using a high-speed burr to allow for adequate room for insertion of a new implant. As previously mentioned, Briant-Evans et al. [16] routinely debulked the distal cement mantle as part of their technique.

We had 1 stem fracture case which occurred in one of the short Exeter stems. Berg et al. [8] presented a case series of 50 short Exeter stems (44/00/125) used in c-in-c revisions, none of which were performed for periprosthetic fracture, and had 1 stem fracture (2%) in their cohort. Woodbridge et al. [9] presented a series of 166 cases using short Exeter stems for c-in-c revision, and they obtained 1 case of stem fracture which of note occurred in a patient with chronic infection. Tai et al. [18] raised concerns regarding the stem’s risk of fracture due to its slim body and relatively large offset; however, in their 250 case series, they did not have any cases of this. Thompson et al. found the short Exeter stems had a higher rate of fracture than the standard Exeter stems in their unit (1.25% vs 0.15%) [19].

Brew et al. [15] performed a biomechanical study recreating Vancouver B1 periprosthetic fractures and repaired the fracture using the c-in-c technique. They found that after the c-in-c revision, there was a mean fracture torque of 50 Nm, which was 43% of the primary fracture torque averaging at 117 Nm. They describe how this force exceeds what previous biomechanical studies have shown for the forces that are required for activities of daily living, which are reported to be around 24 Nm [[20], [21], [22]]. They found that only minimal cement interposes the fracture site, despite a large fracture site surface area. As a part of our fixation procedure, we also add an extended locked plate and/or a strut allograft bypassing the fracture site and adding rotational stability, which we feel improve the fracture torque further.

Weinrauch et al. [23] performed a biomechanical study for the c-in-c technique and found that bilaminar cement mantles resulted in 15%-20% reduction in shear strength. They proposed that the adhesion of the cement mantles was not due to mechanical interlock but that it involved a diffusion-based molecular interdigitation due to the introduction of the new monomer, resulting in dispersion of the monomer in old PMMA chains.

In our study, we had a significant rate of dislocation of 20%, which all underwent a revision surgery. Three cases were revised to constrained liners, and 1 was revised due to a complication of a stem pullout while reduction was being attempted. While high, this rate is relatively similar to that in other studies on revision hip arthroplasty, with figures being quoted between 10% and 30% [24,25].

The patient cohort in our study was a very vulnerable population reflected in the ASA scores and Charlson Comorbidity Index. We had a very high mortality rate in our group (50%), but given the age demographics and comorbidities in this cohort, it is not unexpected. We would like to note that none of these patients passed away in the immediate postoperative period.

There are a number of limitations in this study including that this was a retrospective study. While the study size was small, this was a relatively large study size for this infrequently performed procedure because of the quite specific indications for performing this procedure. Patient-reported outcomes were not collected. There was no other treatment option assessed that could be used for comparison.

We present ORIF and c-in-c revision as a treatment option in patients with selected Vancouver B periprosthetic fractures around a tapered stem where there is a reducible fracture with a well-fixed cement mantle.

## Conflicts of interest

The authors declare there are no conflicts of interest.

For full disclosure statements refer to https://doi.org/10.1016/j.artd.2022.101071.

## Appendix A Supplementary data

Conflict of Interest Statement for McCarthy

Conflict of Interest Statement for Condon

Conflict of Interest Statement for Tiedt

Conflict of Interest Statement for Moore
